# Rare Cause of Recurrent Hypoglycemia: Insulin Autoimmune Syndrome

**DOI:** 10.1155/2017/6423852

**Published:** 2017-11-26

**Authors:** Rungsima Tinmanee, Rungpailin Buranagan, Sirirat Ploybutr, Raweewan Lertwattanarak, Apiradee Sriwijitkamol

**Affiliations:** Division of Endocrinology and Metabolism, Department of Medicine, Faculty of Medicine Siriraj Hospital, Mahidol University, Bangkok, Thailand

## Abstract

We report a case of insulin autoimmune syndrome associated with several autoantibodies, presenting with recurrent hypoglycemia, predominantly in the postprandial period, which improved by dietary management and spontaneously resolved within two months. Differentiation from other causes of hyperinsulinemic hypoglycemia, such as insulinoma, is important to avoid unnecessary invasive procedures or surgical interventions. The 75-gram oral glucose tolerance test (OGTT) and mixed meal test showed a typical pattern, which may be useful indirect evidence of insulin autoimmune syndrome.

## 1. Introduction

Insulin autoimmune syndrome (IAS) or Hirata disease, a rare cause of hyperinsulinemic hypoglycemia first described by Hirata et al. in 1970 [1], is characterized by concurrent hypoglycemia with a very high insulin level and the presence of insulin autoantibodies in insulin-naïve patients [2]. Several autoimmune diseases, predominantly Graves' disease, are associated with insulin autoimmune syndrome [3–5].

## 2. Case Presentation

A 74-year-old previously healthy woman was referred to our hospital due to palpitation and sweating for one month. She did not take any medication. Her first episode of palpitation, sweating, and sleepiness was two years ago. The symptoms, which mostly occurred four hours after lunch, were improved by syrup intake. No symptom was reported in the morning. She was sent to a local hospital, and the capillary plasma glucose (CPG) during symptoms was 49 mg/dl. Unfortunately, neither plasma glucose nor other critical labs were investigated to confirm or diagnose hypoglycemia at that time. Four to six grams per hour of intravenous dextrose solution could maintain her CPG at the normal level. Whole abdominal computed tomography was performed and no abnormality was detected. After the episode for 1 month, her symptoms spontaneously resolved and no more CPG or fasting plasma glucose was recorded. Being afraid of hypoglycemia, consumption of simple carbohydrate such as fruit juices or syrup was added in the late morning and the afternoon, which resulted in a slow gain, from 50 to 60 kg within 2 years, of body weight. The symptoms have been absent for the past two years. One month ago, the several episodes of palpitation and sweating recurred almost every day without any change in her diet pattern. At this time, the symptoms developed at 3 hours after lunch and alleviated after the afternoon snack ingestion. She denied using hypoglycemic agents, insulin, or any drugs or supplements. No hypothyroid or thyrotoxicosis symptoms were recognized. No fever or rash or alopecia or arthritis was experienced. No anemic symptom or polyuria or bone pain was developed. No family history of diabetes or neuroendocrine tumor was noticed. Physical examination revealed a body weight of 60 kg, a height of 155 cm, a body mass index of 28.5 kg/m^2^, a temperature of 36°C, blood pressure of 140/80 mmHg, a pulse of 60 bpm, and a respiratory rate of 16/min. There was no acanthosis nigricans, lipodystrophy, rash, or needle mark on examination. The thyroid gland was not enlarged. No signs of thyrotoxicosis or systemic lupus erythematosus were revealed. Other findings were within the normal.

Laboratory investigations were performed as follows. Complete blood count showed normocytic anemia with hemoglobin of 9.8 g/dL and normal white blood cell count and platelet count. Electrolyte, kidney, and liver functions were within the normal limit. Capillary plasma glucose was 43 mg/dl, together with venous plasma glucose of 44 mg/dl, during the episode of palpitation and sweating symptoms. Serum insulin level at that time was higher than 1,000 *μ*U/mL, and the level was increased to 2,628 *μ*U/mL after 1 : 20 dilution. During the symptoms, she also had a high level of serum C-peptide (at a level of 8.3 ng/mL) and serum cortisol (at a level of 26.7 *μ*g/dL) and an undetected serum *β*-hydroxybutyrate level (at a level of 0 mmol/L). Therefore, endogenous hyperinsulinemic hypoglycemia was confirmed. Oral glucose tolerance test (OGTT) with 75 grams of glucose was performed. Baseline plasma glucose was 79 mg/dL and serum insulin level was 950.3 *μ*U/mL. Her plasma glucose increased to 221 mg/dL at two hours after OGTT. However, 5 hours after OGTT, she developed hypoglycemic symptoms with the plasma glucose dropped to 36 mg/dL, together with a very high serum insulin level of 2,912 *μ*U/mL. Mixed meal test was also performed with the baseline plasma glucose of 84 mg/dL and insulin level of 1,219.6 *μ*U/mL. Symptomatic hypoglycemia was developed at 4 hours after the meal with concurrent plasma glucose of 53 mg/dL and insulin level of 2,110 *μ*U/mL. Because of postprandial hypoglycemia together with the extremely high level of serum insulin, the autoimmune insulin syndrome was suspected and then was confirmed by the very high level of serum insulin autoantibody, with the level of 10.7 nmol/L (normal range: 0–0.02 nmol/L). Further investigations for other associated autoimmune diseases showed a high level of serum anti-thyroglobulin antibody and serum anti-thyroperoxidase antibody with the level of 88.84 U/mL (normal range < 35 U/mL) and 169.26 U/mL (normal range < 65 U/mL), respectively. She also had a positive titer of fine-speckled pattern antinuclear antibody (ANA) with the titer of 1 : 320 (normal titer < 1 : 100). There were no clinical clues of monoclonal gammopathy or the usage of sulfhydryl group-containing drugs in our patient.

The patient was treated by cessation of snack meals and avoidance of simple carbohydrate diet. Two days after dietary adjustment, her CPG was maintained between 65 and 130 mg/dl without requirement of intravenous dextrose solution. She was discharged without any additional therapies. The overall hypoglycemic duration of this episode was two months before the spontaneous remission. The follow-up laboratory investigations are shown in Table 1. Up to 15 months after the 2nd episode of hypoglycemia, she was fine without any symptoms of hypoglycemia.

## 3. Discussion

Insulin autoimmune syndrome (IAS or Hirata disease) is a rare cause of hyperinsulinemic hypoglycemia. To date, approximately 400 cases have been reported and more than 90% of them were Japanese patients [6]. The causes of IAS are heterogeneous, typically associated with certain HLA class II allele, the usage of sulfhydryl group-containing drugs in which methimazole is responsible for almost half of them, autoimmune diseases, monoclonal gammopathy, or hepatitis C viral infection [7]. Graves' disease was the most common autoimmune disease found in IAS [7], while other associated autoimmune diseases, including Hashimoto's thyroiditis, systemic lupus erythematosus, rheumatoid arthritis, antineutrophil cytoplasmic antibodies-associated glomerulonephritis, polymyositis, systemic sclerosis, and ankylosing spondylitis, were found infrequently [3–5]. The mechanism of endogenous autoantibody induced hypoglycemia is that, after food intake, the endogenous antibodies bind to the secreted insulin and proinsulin(s) causing the insulin to be ineffective and causing postprandial hyperglycemia, consequently increasing insulin release further. The dissociation of insulin from insulin autoantibodies resulted in extremely high levels of unbound insulin and caused hypoglycemia [8, 9]. From a previous case report, about 42% had postprandial hypoglycemia, 31% had fasting hypoglycemia, and 24% had both postprandial and fasting hypoglycemia. The majority of patients are above 40 years of age, and women and men are affected equally [10].

Because of the typical postprandial hypoglycemia and very high insulin level without signs of insulin resistance, insulin autoimmune syndrome was suspected in our patient. The significantly elevated insulin autoantibodies level in insulin-naïve patients confirmed the diagnosis of insulin autoimmune syndrome. While the result of insulin autoantibodies was pending, we did not further investigate the unnecessary invasive procedures or surgical intervention in our patient. The age onset of 74 years old was around the peak age of onset in Japanese case series [11]. We assumed that the first episode of IAS with spontaneous remission occurred two years ago, and this episode was her 2nd attack. Uchigata et al. [11] reported that 82% of the patients in Japan had a spontaneous remission of IAS within 3–6 months, with the peak duration more than 1 and less than 3 months [11]. Our patient also experienced a three-month duration in the first episode and two-month period in the last episode. Thyroid autoantibodies and antinuclear antibody (ANA) were detected in the patient. Elevated ANA titer and thyroid autoantibodies, in the period of hypoglycemic attack, were decreased after the remission of disease along with the declination of fasting insulin and improvement of low plasma glucose. These findings support the association of IAS and autoimmunity. The upward changing trends of autoantibodies in the follow-up period may be the early indicator of relapsing disease.

The 75-gram OGTT in our patient showed a diabetic pattern at two hours with plasma glucose of 221 mg/dl, corresponding to the diabetic pattern of 59% in autoimmune insulin syndrome patients reported by Uchigata et al. [2]. The ineffective insulin that bound with their autoantibodies as previously described can explain the mechanism of diabetic or impaired glucose tolerance pattern. Hypoglycemia developed at five hours after administration of 75 grams of glucose, one hour later compared with the result of Lupsa et al. [10]. In the mixed meal test, hypoglycemia developed at four hours, similar to the result from one of the patients from the study of Lupsa et al. [10]. In our patients, hypoglycemia was more severe, indicated by lower plasma glucose, together with the higher level of insulin after 75-gram OGTT than after mixed meal test; this could imply that the intake of simple carbohydrate can aggravate severe hypoglycemia.

A previous study by Uchigata and Hirata [7] in 330 Japanese IAS patients demonstrated that 78% of the patients had spontaneous remission or resolution after avoiding culprit drug exposure, 9% required steroid treatment, 7.3% were treated with alpha-glucosidase inhibitor, 2.4% had severe hypoglycemia and required plasmapheresis, 1.8% underwent pancreatic surgery, and 0.6% were treated with other immunosuppressive drugs such as azathioprine and 6-mercaptopurine. Our patient was successfully treated with dietary management mainly by avoidance of simple carbohydrate. Hypoglycemia spontaneously remitted within two months.

## 4. Conclusion

We reported a case of recurrence postprandial hypoglycemia with an extremely high insulin level leading to the diagnosis of insulin autoimmune syndrome, which was confirmed by the high level of serum insulin autoantibody. With this clinical syndrome, we can diagnose the cause of hypoglycemia in this patient and prevent her from undergoing unnecessary investigation and surgery.

## Figures and Tables

**Table 1:** Laboratory investigation during initial and subsequent follow-up period.

Lab	Time after the 2nd episode				
	1st day	4th month	6th month	12th month	15th month
FPG (mg/dL)	44^*∗*^	96	87	93	92
HbA1c (%)	5.7	5.7	5.8	5.7	5.8
Fasting insulin level (*µ*U/mL)	2628^*∗*^	50.77	26.11	18.17	12.62
C-peptide level (ng/mL)	8.3^*∗*^	—	1.95	2.23	2.16
Insulin autoantibody level (nmol/L)	10.7^*∗*^	—	—	—	—
Anti-thyroperoxidase Ab (U/mL)	88.84	—	62.10	43.35	35.11
Anti-thyroglobulin Ab (U/mL)	169.26	—	114.11	68.11	72.40
Antinuclear Ab titer	1 : 320	—	1 : 320	1 : 100	—

^*∗*^Results obtained during the hypoglycemic episode.
